# Comparative Transcriptome Analysis of *Pinus densiflora* Following Inoculation with Pathogenic (*Bursaphelenchus xylophilus*) or Non-pathogenic Nematodes (*B. thailandae*)

**DOI:** 10.1038/s41598-019-48660-w

**Published:** 2019-08-21

**Authors:** Il Hwan Lee, Hyerim Han, Young Ho Koh, In Sik Kim, Seok-Woo Lee, Donghwan Shim

**Affiliations:** 1 Department of Forest Bio-Resources, National Institute of Forest Science, Suwon, 16631, Republic of Korea; 2 Division of Forest Insect Pests and Diseases, National Institute of Forest Science, Seoul, 02455, Republic of Korea; 3 Ilsong Institute of Life Science, Hallym University, Anyang, Republic of Korea

**Keywords:** Biotic, Plant molecular biology

## Abstract

*Pinus densiflora* (Korean red pine) is a species of evergreen conifer that is distributed in Korea, Japan, and China, and of economic, scientific, and ecological importance. Korean red pines suffer from pine wilt disease (PWD) caused by *Bursaphelenchus xylophilus*, the pinewood nematode (PWN). To facilitate diagnosis and prevention of PWD, studies have been conducted on the PWN and its beetle vectors. However, transcriptional responses of *P. densiflora* to PWN have received less attention. Here, we inoculated Korean red pines with pathogenic *B. xylophilus*, or non-pathogenic *B. thailandae*, and collected cambium layers 4 weeks after inoculation for RNA sequencing analysis. We obtained 72,864 unigenes with an average length of 869 bp (N50 = 1,403) from a Trinity assembly, and identified 991 differentially expressed genes (DEGs). Biological processes related to phenylpropanoid biosynthesis, flavonoid biosynthesis, oxidation–reduction, and plant-type hypersensitive response were significantly enriched in DEGs found in trees inoculated with *B. xylophilus*. Several transcription factor families were found to be involved in the response to *B. xylophilus* inoculation. Our study provides the first evidence of transcriptomic differences in Korean red pines inoculated with *B. xylophilus* and *B. thailandae*, and might facilitate early diagnosis of PWD and selection of PWD-tolerant Korean red pines.

## Introduction

Pines are conifers in the genus *Pinus* that are found in the Northern Hemisphere^1^. They have economic, ecological, and scientific importance, as they provide timber for construction, furniture, paneling, and flooring^2,3^, habitats and food for wildlife, and (in their needles) agents with anticancer, antioxidant, and antimutagenic properties^4^. However, pine trees (such as *Pinus densiflora*, *P. thunbergii*, and *P. koraiensis*) are threatened by pine-wilt disease (PWD), a devastating disease that kills trees within a few weeks to a few months from infection, with symptoms that are characterized by wilted and brown-colored needles^5,6^. PWD is caused by the pinewood nematode (PWN) *Bursaphelenchus xylophilus*, which is carried by long-horned beetles (*Monochamus* spp.) and spread when the beetles feed on the trees^5^. *B. thailandae* is a nematode species that was first isolated from pine trees in Thailand, and which has subsequently been detected in Korea. *B. thailandae* differs morphologically from *B. xylophilus*, and is not pathogenic, so pine trees inoculated with *B. thailandae* can survive without showing any visible symptoms of PWD^7,8^. Therefore, examination of the transcriptome differences between *B. xylophilus* and *B. thailandae* inoculated pines might identify novel resistance mechanisms and candidate genes that play important roles in resistance specifically against *B. xylophilus*. Furthermore, we also might identify the pathogenesis mechanisms of *B. xylophilus* to cause PWD. However, comparative transcriptome analysis between trees inoculated with nematodes having different pathogenicity has not been conducted.

Many studies have been carried out to find ways to diagnose and block PWD, and most of them have been focused on *B. xylophilus* and its beetle vectors^9–11^. Currently, to diagnose PWD, *B. xylophilus* or DNA fragments from *B. xylophilus* must be detected in tree samples^12^, or PWD symptoms must be observed. However, it is difficult to detect *B. xylophilus* and its DNA in pine trees at an early stage of infection, and by the time PWD symptoms can be observed, *B. xylophilus* has generally already spread throughout the forest.

The physiological symptoms of PWD have been well characterized, but there are only few studies that attempt to understand the comprehensive transcriptome of pine trees in response to *B. xylophilus* infection. Previously, we examined the transcriptome differences between trees with and without PWD symptoms in natural forest. However, we didn’t know whether trees showing PWD symptoms are indeed infected by PWN or not^13^. In this report, we inoculated trees with PWN and observed the PWD symptoms after inoculation of PWN. Therefore, we can assure that PWD symptoms are indeed caused by PWN inoculation, and examination of the transcriptome differences among the trees injected by water, non-pathogenic nematode, and pathogenic pine wood nematode give better understanding of the transcriptional responses against PWD in Korean red pines.

In the thale cress *Arabidopsis thaliana*, thousands of genes have been shown to be differentially expressed upon nematode infection, and important regulators of defense responses against nematode infection have been discovered by transcriptomic analysis^14,15^. By extension, it might also be possible to identify genes that are differentially expressed in pine trees upon infection with *B. xylophilus*, and thereby to diagnose PWD infection by gene-expression analysis. In addition, it might be possible to develop PWD-resistant trees by selection of particular alleles of genes that are differentially expressed after inoculation with *B. xylophilus*.

Despite the importance of developing a comprehensive understanding of the transcriptome of pines in conditions of *B. xylophilus* infection, there is a lack of transcriptome analysis and most of them were conducted using saplings under artificial experimental conditions instead of using adult trees in a forest environment^16,17^. Next-generation sequencing (NGS) technology has been rapidly developed and widely used for research in plant biology, to enhance our understanding of plant responses under various conditions^18^. In addition, software developments now enable the *de novo* assembly of the transcriptome of an organism (such as *P. densiflora*) that does not have a reference transcriptome^19^.

Here, we report the *de novo* assembly of the *P. densiflora* transcriptome and quantification of transcript expression in response to inoculation with either *B. xylophilus* or *B. thaliandae* at felling age in a natural forest environment. Identification of differentially expressed transcripts in pathogenic PWN-inoculated *P. densiflora* could spur the development of a diagnostic method for PWD infection and aid in the selective breeding of PWD-resistant *P. densiflora*.

## Materials and Methods

### Plant materials and inoculation with PWN

Pathogenic PWN (*B. xylophilus*) and non-pathogenic nematode (*B. thailandae*) were originally isolated from Korean red pines and reared on fungal hyphae of *Botrytis cinerea* (de Bary) Whetzel grown on potato dextrose agar medium at 25 °C for 2 weeks. Nematodes were re-isolated from the medium by the Baermann funnel method^20^. Nine Korean red pines of 11–13 m height and 15–20 cm diameter at breast height in a forest in Jinju-si, Gyeongsangnam-do province, South Korea, were selected. Water, *B. xylophilus*, and *B. thailandae* were injected into three independent trees each. The stem of each tree at breast height was wounded mechanically in three places, and 1 ml sterile water or 1 ml sterile water containing 20,000 nematodes was injected into each wound site (for a total of 60,000 PWNs per tree). Cambium samples of the trees were collected 4 weeks after inoculation and subjected to RNA-Seq analysis. Briefly, hard outer bark was removed and soft cambium layers were taken from the main stem at breast height by using chisel.

### RNA extraction, cDNA library preparation, and sequencing

Cambium samples were taken from the main stem at breast height by using chisel. Total RNA was isolated from cambium samples with an RNA isolation kit (TAESIN Bio Co., Seoul, South Korea). The assessment of RNA integrity (RIN), library construction, and sequencing were performed as described previously^13^. Briefly, RNA quality was determined with a 2100 Bioanalyzer (Agilent, Santa Clara, CA, USA), and only samples with an RNA integrity number >8 were used for library preparation. Preparation of each paired-end non-directional cDNA library (2 × 101 bp) was conducted according to the TruSeq RNA Sample Preparation Guide (Illumina, San Diego, CA, USA). Sequencing of cDNA libraries was performed on an Illumina HiSeq. 2000 sequencer.

### *De novo* transcriptome assembly of nematode inoculated *P. densiflora*

PRINSEQ-lite v0.20.4 was used for read cleaning as described in Lee *et al*. (2018) with minor modification (filtering of sequences <50 bp length and eliminating exact duplicates or reverse-complement exact duplicates caused by library PCR amplification with –derep 14 option)^13,21^. Reference transcriptome were generated from all clean reads using Trinity v2.5.1 with default parameters^19^ and *de novo* assembled transcriptome is available as Supplementary Data S1. Candidate coding regions in the all assembled transcripts were identified using TransDecoder v5.3.0 with default parameters (two steps such as extraction of the long ORFs and prediction of the likely coding regions)^22^. Clustering of transcripts was performed using CD-HIT-EST v4.6.1 with default parameters^23,24^, and the longest transcripts in each cluster were used for subsequent analysis. Transcriptome completeness was assessed by using Benchmarking Universal Single-Copy Orthologs (BUSCO) v3 with the Embryophyta_(odb10) database^25^.

### Quantification of the expression of transcripts and identification of differentially expressed transcripts

Clean paired end reads were mapped to the reference transcriptome using Bowtie software^26^. Read counts were obtained using RSEM v1.3.0^27^. The raw counts were normalized as trimmed mean of M-values (TMM)-normalized transcripts per kilobase million (TPM) values for each transcript and differentially expressed genes (DEGs) showing more than 2-fold expression change with a false-discovery rate (FDR)-adjusted *P*-value ≤ 0.05 among all pairwise sample comparisons were obtained using EdgeR v3.16.5, a component of Trinity^19,28^.

### Gene annotation, gene ontology enrichment, and MapMan analysis of DEGs

Translated protein sequences corresponding to assembled unigenes were compared with those of *A. thaliana*, with an E-value ≤ 1E-7 using the Basic Local Alignment Search Tool for proteins (BLASTX) with default parameters except for max_target_seqs 1^29^ and annotation result was presented as Supplementary Data S2. Putative *P. densiflora* transcription factors (TFs) that aligned and annotated with *A. thaliana* TFs were classified into TF families based on the Plant Transcription Factor Database v4.0 (http://planttfdb.cbi.pku.edu.cn/)^30^. Gene ontology (GO) enrichment analysis was performed with PANTHER GO classification system (http://www.geneontology.org)^31^. Gene ontology information files from The Arabidopsis Information Resource (TAIR) were used as a reference^32^. Biological process gene ontology (GOBP) with a Fisher’s exact test with FDR corrected *P*-value va0.05 were considered to be significantly enriched. REVIGO (http://revigo.irb.hr) were used for reducing and visualizing the genes ontology^33^. For MapMan analysis, homologous *A. thaliana* IDs and log_2_ fold-change values of TMM normalized TPM (*B. xylophilus* versus *B. thailandae*) were mapped to biotic-stress pathways^34^. Pictorial representation for the biotic-stress pathways was downloaded from the MapMan website (https://mapman.gabipd.org/)^34^.

### Correlation analysis between quantitative PCR and NGS data

Preparation of total RNA from Korean red pines was performed using RNA isolation kit (TAESIN), and quantitative reverse-transcription PCR (RT-qPCR) was conducted using the TOPreal One-step RT qPCR Kit (Enzynomics, Daejeon, South Korea). 12 DEGs in trees inoculated with *B. xylophilus* relative to those inoculated with *B. thailandae* were used for RT-qPCR with a CFX96 Touch Real-Time PCR Detection System (Bio-Rad, Hercules, CA, USA). Expression levels of the genes were calculated by the comparative threshold method, with *EIF4A-2* as the internal control. The Pearson correlation coefficient between RT-qPCR and RNA-Seq was analyzed with the R statistical software^35^. Primer sequences are listed in Supplementary Table S1 and a related script for correlation analysis in R were presented in Supplementary Data S3.

### Data deposition

All the raw read sequences were deposited in the NCBI sequence read archive under the accession number SRP165817.

## Results

### Visible PWD symptoms in nematode-inoculated Korean red pines

**Figure 1:**
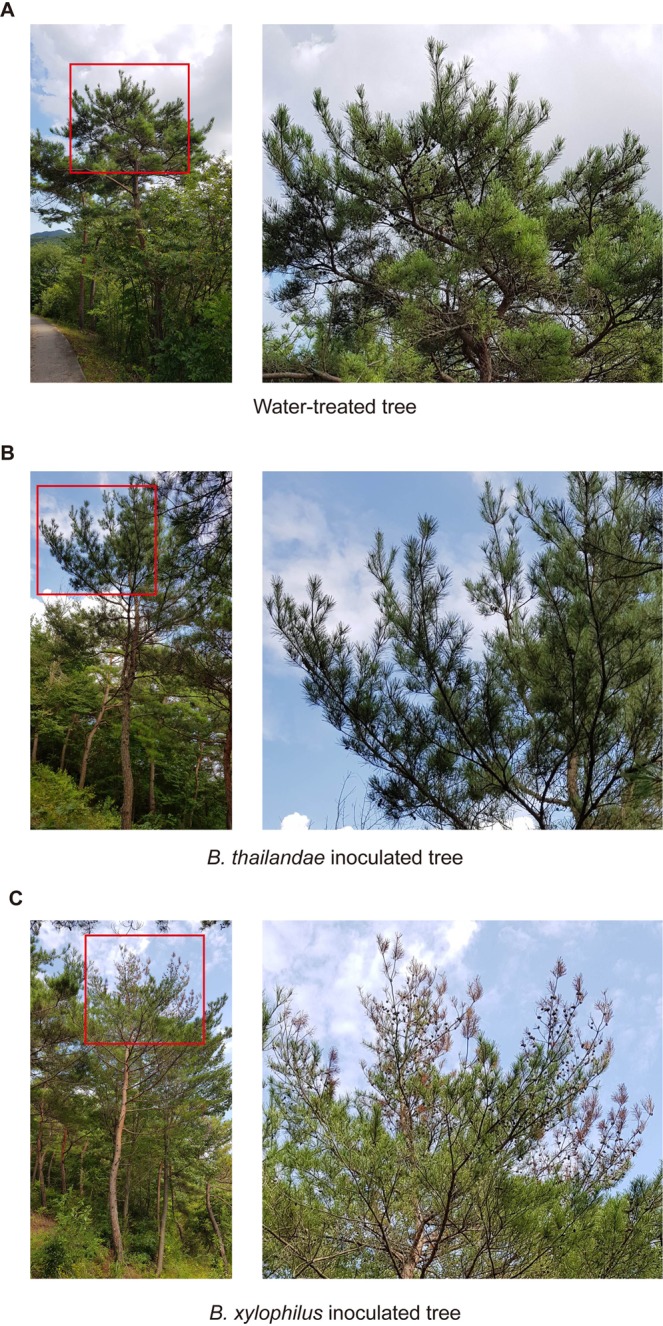
Physiology of Korean red pines 3 months after inoculation with water, *Bursaphelenchus thailandae*, or *B. xylophilus*. Mature trees were wounded and injected with water or with suspensions of 60,000 nematodes per tree. Physiology of the trees was inspected 3 months after treatment. Representative images of trees injected with water (**A**), *B. thailandae* (**B**), and *B. xylophilus* (**C**) are shown. Higher magnification images of the regions indicated by red squares are displayed in the right-hand panels.

To examine the relative pathogenicity of two species of the genus *Bursaphelenchus*, we monitored the visible phenotype of selected *P. densiflora* trees injected with water, *B. xylophilus*, or *B. thailandae*. None of these trees showed any visible PWD symptoms 4 weeks after treatment. However, 3 months after treatment, some of the green needles turned to brown and drooped in trees inoculated with *B. xylophilus*, whereas trees treated with water or *B. thailandae* still displayed no visible PWD symptoms (Fig. 1). This result is consistent with the previous observation that *B. xylophilus* is pathogenic, whereas *B. thailandae* is non-pathogenic.

### *De novo* transcriptome assembly of nematode-inoculated Korean red pines

To examine the transcriptional programs in response to inoculation with B. xylophilus or B. thailandae, we sampled cambium layers of trees 4 weeks after treatment and conducted RNA-Seq, generating 271,466,695 raw paired reads (54,836,272,390 bp). Finally, 213,695,875 cleaned paired reads were used for *de novo* assembly of *P. densiflora* transcriptome (Supplementary Table S2). In total, 72,864 unigenes (161,111,300 nucleotides) were generated by using the Trinity assembler^19^ (Table 1). BUSCO analysis showed that 87.4% (1,166 single copy genes and 36 duplicated genes) complete BUSCO genes were presented in the transcriptome. In addition, 6.3% (86 genes) of all BUSCO genes were presented as fragmented form and 6.3% (87 genes) were missing in the *de novo* assembled transcriptome, respectively (Supplementary Fig S1). Candidate coding regions were predicted within the transcripts and the number of each type of transcripts was listed in Supplementary Table S3 and sequencing depth for both transcripts and unigenes were also presented in Supplementary Table S4.

**Table 1:** Statistics from *de novo* assembly of transcripts in *Pinus densiflora*.

Assembled contigs	
Total Trinity genes, *n*	72,864
Total Trinity transcripts, *n*	185,501
Percent GC	42.44
Contig N50 length (bp)	1,403
Average contig length (bp)	869
Contig E90N50 length (bp)	2,020
E90 number of transcripts	15,645
Total assembled bases	161,111,300

### Identification of DEGs in response to nematode inoculation in Korean red pines

**Figure 2:**
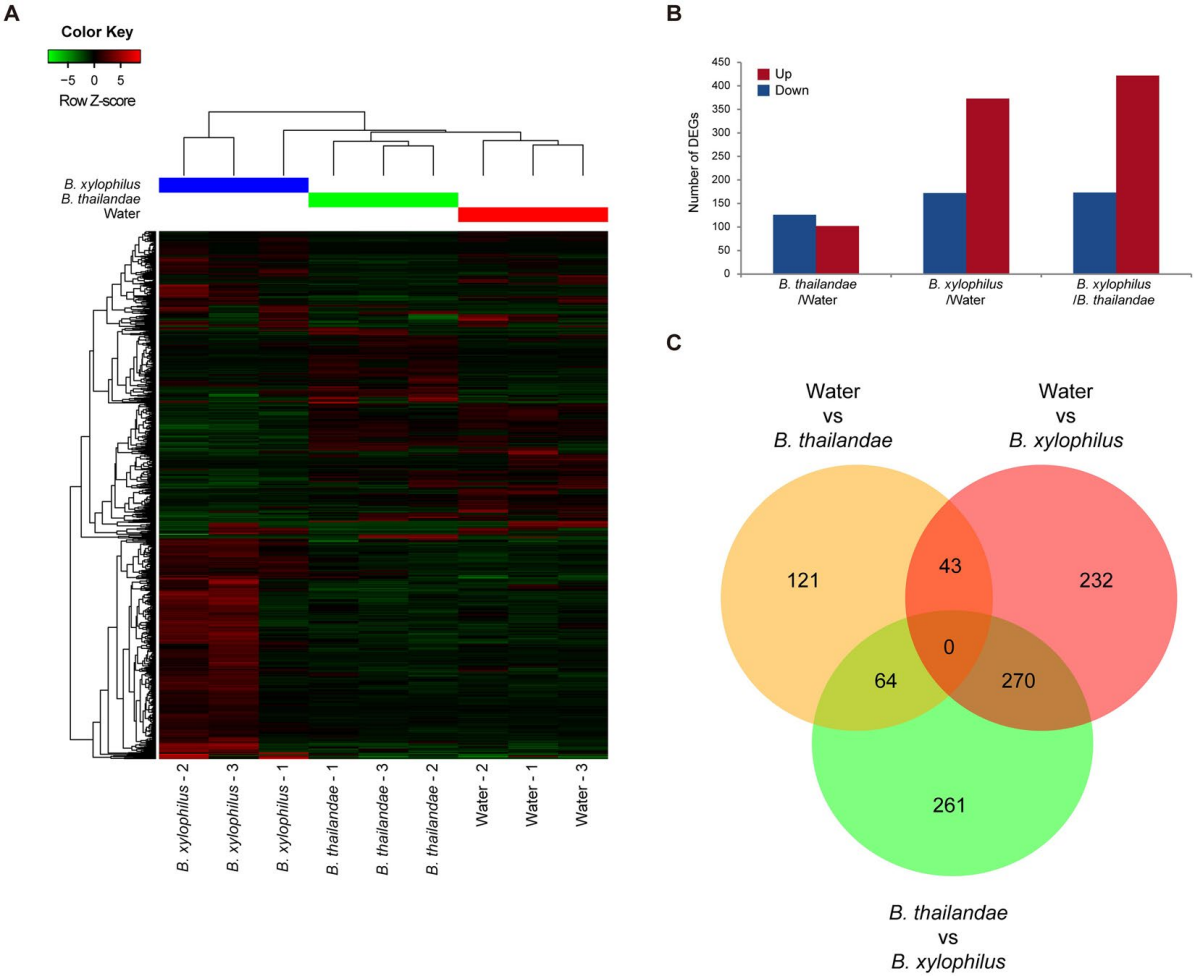
Identification of differentially expressed genes (DEGs) in Korean red pine trees treated with water, *Bursaphelenchus thailandae*, and *B. xylophilus*. (**A**) Heatmap of relative expression of 991 DEGs in nine trees with the indicated treatments. Expression values are log2-transformed median-centred TMM-normalized TPM. Color Key indicates Row Z-scores of expression values. The x-axis dendrogram indicates sample similarity and y-axis dendrogram indicates the hierarchical clustering of unigenes with similar expression profiles. Distance and clustering algorithms used for the dendrogram were complete linkage with Euclidean distances. (**B**) Number of genes showing up- or down-regulated expression in pairwise comparisons. (**C**) Venn diagrams indicating the numbers of DEGs for each comparison.

At first, we examined the correlation of the biological samples to investigate relationships among them. PCA and correlation heatmap showed that water injected tree - 3 (water - 3), *B. thailandae* inoculated tree - 1 (*B. thailandae* - 1) and *B. xylophilus* inoculated tree - 1 (*B. xylophilus* - 1) were not closely clustered together with their biological replicates (Supplementary Fig. S2). In this experiment, we inoculated and sampled the trees in forest. Therefore, experimental condition (light, temperature, watering, etc.) is not under control. In addition, the trees are not genotypically identical (not clones). These might cause different responsiveness to inoculation of PWN and subsequent transcriptome differences within the biological replicates. In nature, genetic and phenotypic heterogeneity is commonly observed thus we used all the biological samples to reflect the real ecological system for further transcriptome analysis. To identify the genes that are related to response against nematode inoculation in Korean red pines, we examined differentially expressed genes among the comparisons using the three biological replicates each (Supplementary Fig. S3). In total, 991 genes were differentially expressed (with a cutoff of greater than 2-fold change with a *P*-value for FDR <0.05) in all the pairwise comparisons (Fig. 2A). Among the DEGs, 102 were significantly up-regulated and 126 were down-regulated in trees inoculated with *B. thailandae*, compared with injection of water only. In trees inoculated with *B. xylophilus*, 373 DEGs were up-regulated and 172 were down-regulated, compared with water-only controls. Comparison of the transcriptomes of trees inoculated with *B. xylophilus* or *B. thailandae* identified 595 transcripts as reliable DEGs. Among them, 422 were up-regulated and 173 were down-regulated in trees inoculated with *B. xylophilus* compared with trees inoculated with *B. thailandae* (Fig. 2B and Supplementary Fig. S4). Intriguingly, no unigenes were identified as reliable DEGs that commonly observed in all pairwise comparisons. In addition, the largest number of unigenes (270) was commonly identified as DEGs in the comparison between water and *B. xylophilus*, and *B. thaliandae* and *B. xylophilus* injected trees (Fig. 2C). The Venn diagrams for up- and down-regulated genes are separately presented in Supplementary Fig. S5.

### GO enrichment analysis for DEGs

**Figure 3:**
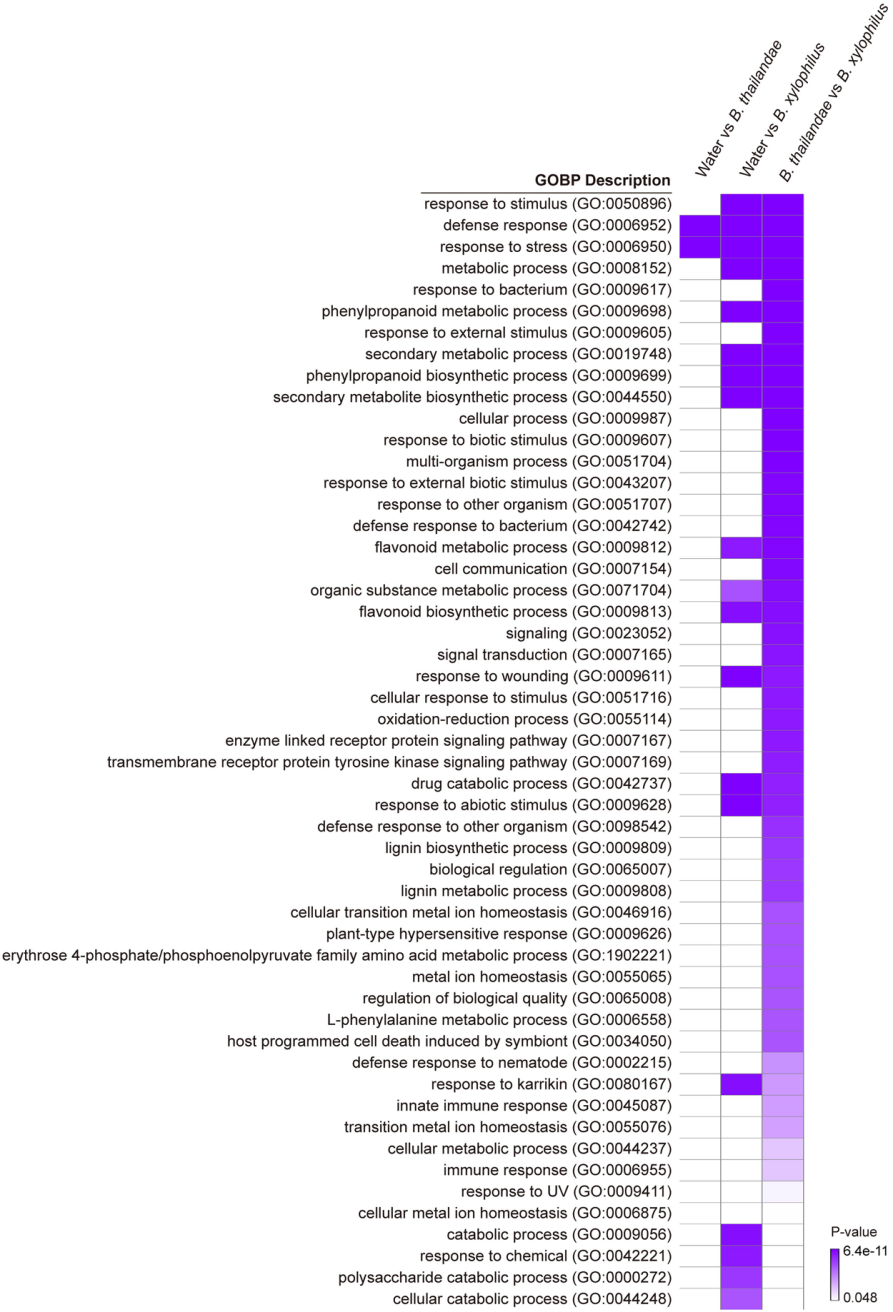
Heatmap of gene ontology (GO) analysis of differentially expressed genes in comparisons between Korean red pine trees treated with water, *Bursaphelenchus thailandae*, and *B. xylophilus*. The heatmap shows the GO biological process (GOBP) terms associated with the indicated comparisons (with Fisher’s exact test with FDR corrected *P*-value < 0.05). Color Key indicates Fisher’s exact test with FDR corrected *P*-value.

To obtain comprehensive functional features associated with transcriptional programs in response to *B. xylophilus* inoculation, we categorized the DEGs on the basis of Gene Ontology Biological Processes (GOBPs) (Fig. 3 and Supplementary Fig. S6). GOBP analysis was conducted for the best hits from the *Arabidopsis* genome. GOBPs related to defense response and response to stress were enriched in the DEGs identified in the comparison between trees inoculated with *B. thailandae* or treated with water. In the comparison between trees inoculated with *B. xylophilus* and those treated with water, in addition to terms shared with other comparisons (such as the defense response and response to stress, and phenylpropanoid and flavonoid biosynthetic processes), the GOBP terms catabolic process, response to chemical, polysaccharide catabolic process, and cellular catabolic process were specifically enriched. Notably, the largest number of GOBPs was enriched in the DEGs identified in the comparison between trees inoculated with *B. xylophilus* and *B. thailandae*. Among these terms, response to bacterium, cell communication, oxidation–reduction process, transmembrane receptor protein tyrosine kinase signaling pathway, lignin biosynthetic process, plant-type hypersensitive response, defense response to nematode, and innate immune response were specific to this comparison. Heatmap of GOBPs enriched in the DEGs in each category of Venn diagram in Fig. 2C was shown in Supplementary Fig. S7.

### Identification of TFs involved in the response to inoculation with *B. xylophilus*

**Figure 4:**
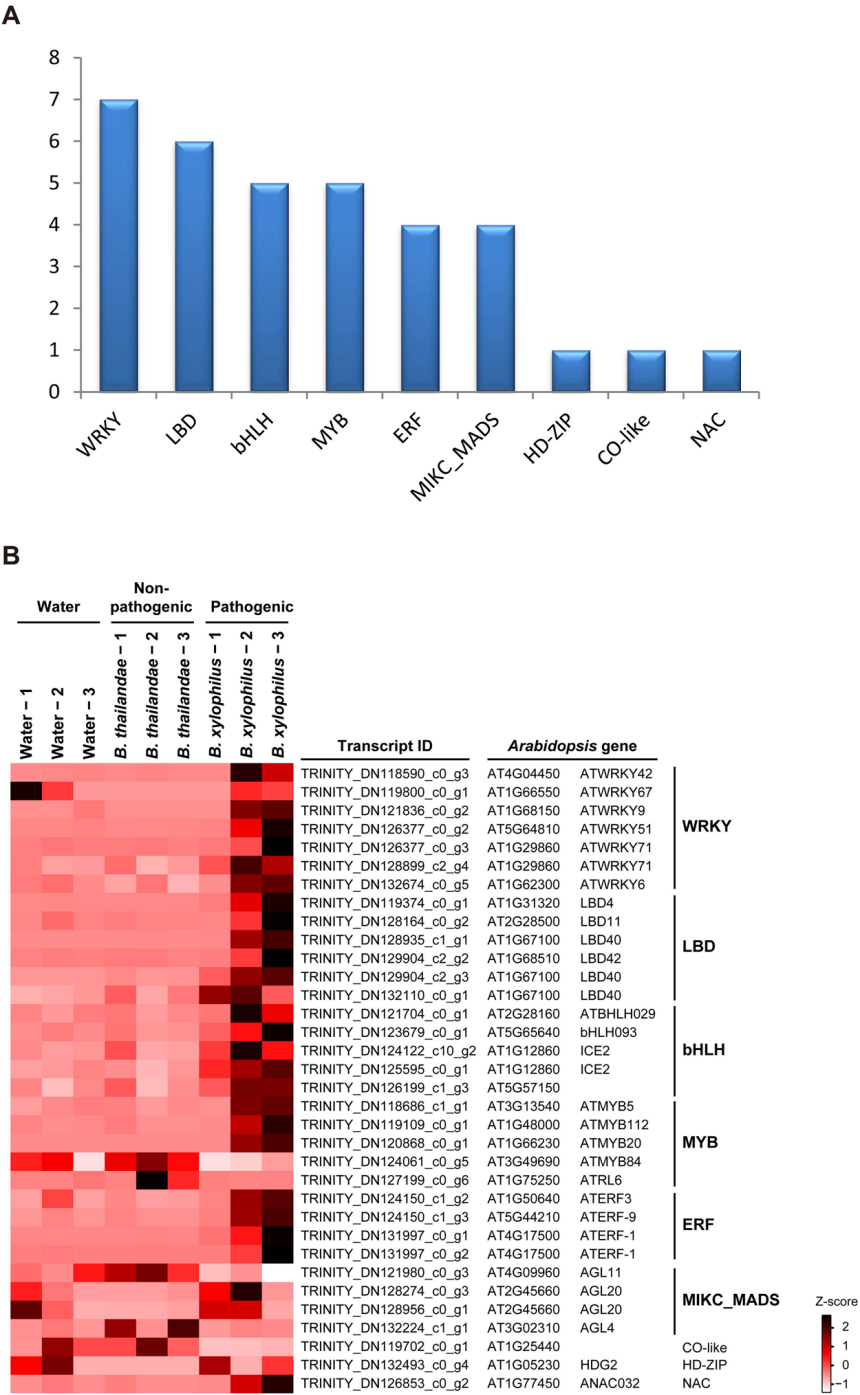
Differential expression of transcription factor (TF) genes in comparisons between Korean red pine trees treated with water, *Bursaphelenchus thailandae*, and *B. xylophilus*. (**A**) Pie chart of the number of TFs in particular families with differential expression in the comparison between trees infected with *B. thailandae* and *B. xylophilus*. (**B**) Heatmap of expression levels of differentially expressed TFs in nine trees with the indicated treatments. Heatmap colors represent the Z-scores of TMM normalized TPM values. The best hits in the *Arabidopsis thaliana* protein database (and the relevant TF families) are shown on the right-hand side of the transcript IDs.

TFs govern transcriptional programs through regulation of expression of target genes. To investigate the regulation of transcriptional programs in Korean red pines in response to *B. xylophilus* infection, we identified differentially expressed TFs (DETFs) in the comparison between trees inoculated with *B. xylophilus* or with *B. thailandae*. We identified 34 TFs as DETFs and the most highly represented DETF families were the WRKY (7 members) followed by LBD (6 members), bHLH and MYB families (5 members each) (Fig. 4A and Supplementary Table S5), and the expression patterns of these TFs in the different cambium samples are shown in Fig. 4B. Many of the individual DETFs were up-regulated in trees inoculated with *B. xylophilus*, compared with their expression in trees inoculated with *B. thailandae*. This result suggested that these DETFs might have important roles in controlling transcriptional programs in response to inoculation with *B. xylophilus* by activating or repressing expression of target genes through binding to cis-acting elements.

### Key elements of biotic-stress pathways are involved in the response to *B. xylophilus*

**Figure 5:**
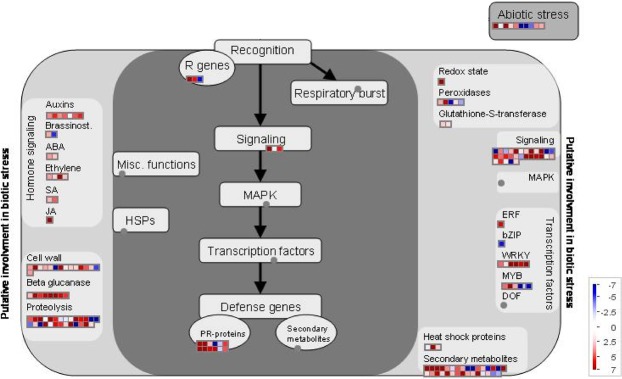
MapMan visualization of *Pinus densiflora* genes involved in the response to *Bursaphelenchus xylophilus* inoculation of Korean red pine trees. Overview of expression levels of DEGs (log_2_ fold-change of TMM-normalized TPM values) in Korean red pines with *B. xylophilus* inoculation relative to trees with *B. thailandae* inoculation. Square dots represent different paralogous genes encoding proteins that are related to a particular defense-response step in *Arabidopsis thaliana*. Red dots indicate up-regulation and blue dots down-regulation. ABA, abscisic acid; brassinost., brassinosteroid; HSP, heat-shock protein; JA, jasmonic acid; PR, pathogenesis-related; SA, salicylic acid.

To investigate key signaling elements in biotic-stress pathways, we identified DEGs in the comparison between trees inoculated with *B. xylophilus* or with *B. thailandae*, and located each gene (and its log_2_ fold-change value) in biotic-stress pathway with the MapMan visualization software (Fig. 5). Genes involved in signaling of phytohormones (such as auxin, abscisic acid, ethylene, salicylic acid, and jasmonic acid) were more highly expressed in response to *B. xylophilus* than to *B. thailandae*. Genes related to cell-wall modification and proteolysis were differentially expressed, and many of them showed elevation of expression in response to *B. xylophilus*. In addition, several genes for pathogen recognition, signaling, and defense response (PR proteins) were more highly expressed in response to *B. xylophilus* than to *B. thailandae*. Oxidation-related processes make up one of the most important pathways in the control of defense responses in plants^36^, and genes related to oxidation–reduction processes were also differentially expressed, as were a number of TFs, including several members of both the WRKY and MYB families. These types of TFs are known to have important roles in regulation of the defense response against pathogen infection^37–40^. The MapMan metabolism and regulation overviews are also shown as Supplementary Fig. S8 and S9, respectively. These results suggest that the biotic-stress pathways and related components identified here are involved in control of the defense response to *B. xylophilus* infection.

### qRT-PCR conformation of expression levels of DEGs

To validate transcriptome results, we conducted qRT-PCR analysis with 12 DEGs in trees inoculated with *B. xylophilus* relative to those inoculated with *B. thailandae*. The expression changes of the DEGs from qRT-PCR and RNA-seq analyses were highly correlated (Supplementary Fig. S10), and it indicated the reliability of the RNA-seq results.

## Discussion

In this study, we inoculated mature Korean red pines with *B. xylophilus* or *B. thailandae* and performed transcriptome analysis to comprehensively understand the responses of trees against pathogenic nematodes. We identified novel resistance mechanisms and candidate genes that play important roles in resistance specifically against *B. xylophilus*. Furthermore, we also tried to identify the pathogenesis mechanisms of *B. xylophilus* to cause PWD. Overall, 991 DEGs were identified, and *B. xylophilus* infection resulted in 595 DEGs compared with *B. thailandae* infection, and 545 DEGs compared with water injection. Notably, *B. thailandae* inoculation only resulted in 228 DEGs compared with water injection, which suggests a limited transcriptional response of the trees to the low pathogenicity of *B. thailandae*.

The physiological nature of the responses to nematode infection was examined by GOBP analysis. Notably, the defense response GOBP was enriched in the DEGs observed in all pairwise comparisons of treatments, suggesting that both *B. xylophilus* and *B. thailandae* commonly affected expression of the genes involved in the defense response, regardless of their pathogenicity in the inoculated trees.

To identify GOBPs related to different extents of PWD in trees infected with different species of nematodes, we examined the genes that were differentially expressed between trees inoculated with *B. xylophilus* and *B. thailandae*. Phenylpropanoid and flavonoid biosynthetic processes were enriched in the DEGs in this comparison. The involvement of phenylpropanoids in the defense response is a well-established phenomenon^41^. Flavonoids, isoflavonoids, hydroxycinnamic acids, monolignols, and stilbenes are types of phenylpropanoids that function as defensive molecules, acting as physical barriers and signaling molecules to induce the defense response against pathogen invasion^42^. These results suggested that expression of genes related to phenylpropanoid biosynthesis is regulated for defense against *B. xylophilus* infection.

We found that the lignin biosynthetic process GOBP was enriched in the DEGs in the comparison between trees inoculated with *B. xylophilus* and *B. thailandae*, and it is well known that lignin is rapidly deposited after nematode invasion, and serves as a mechanical barrier^43^. Correlation between the increase of lignin content and resistance to nematodes, and an influence of lignin composition on nematode resistance, has been observed in several plant species^44^. Previous report showed that lignin concentration had significantly increased after infestation of PWN at an early stage of the infestation in *P. abies* and *C. lusitanica*^45^. In addition, Ishida *et al*. also showed that inoculation of *B. xylophilus* to Japanese black pine caused accumulation of lignin around the resin canals in the cortex^46^. These results could support the validity of our analysis. We also found that expression of genes related to the hypersensitive response and oxidation–reduction process was affected by *B. xylophilus* inoculation. The hypersensitive response is a type of cell death that is associated with plant resistance to pathogen infection^47^. This localized cell death blocks the migration of the pathogen to adjacent cells, and hypersensitive response-associated resistance is also observed in nematode–plant interactions^48^. Plants produce reactive oxygen species (ROS) upon nematode infection, thereby activating defense responses, and ROS are closely related to the hypersensitive response^36,49^. However, ROS also function as pathogenicity factors to facilitate nematode infection in *A. thaliana*^50,51^. Therefore, it is also possible that *B. xylophilus* modulates expression of host oxidation–reduction-related genes to suppress plant defense responses.

In our comparison between trees inoculated with *B. xylophilus* and *B. thailandae*, transmembrane receptor protein tyrosine kinase signaling pathway GOBP were enriched in the DEGs. An effective plant defense against pathogens is achieved through recognition of pathogen-associated molecular patterns by surface-localized receptor kinases, and by the consequent downstream signaling cascade^52,53^. In this analysis, homologues of *Arabidopsis FLS2* and *NILR1* were identified as DEGs in this biological process. Both of them were well known as important regulators in PAMP triggered immunity. In *A. thaliana*, leucine-rich repeat receptor-like kinase NILR1 is required for innate immunity against parasitic nematodes^54^. Therefore, interaction between nematode-associated molecular patterns and transmembrane receptor protein tyrosine kinases might be important for successful resistance to *B. xylophilus* in Korean red pines.

We explored the expression patterns of TFs that mainly govern transcriptional programs responding to *B. xylophilus* infection by comparing the expression of TFs between trees inoculated with *B. xylophilus* and with *B. thailandae*. The family of DETFs with the most members represented by DEGs was WRKY, followed by theLBD, bHLH, MYB, ERF, and MIKC_MADS families. WRKY TFs are involved in many developmental processes, especially in defense and senescence^37^. *AtWRKY6* homologue was one of the DETF genes in the present study, and *AtWRKY6* is a positive regulator in the defense response against the beet-cyst nematode *Heterodera schachtii*, and induces expression of salicylic acid-dependent defense-response genes in *A. thaliana*^38,55^. In addition, *AtWRKY51* homologue was also induced after *B. xylophilus* inoculation, and it is reported that *WRKY51* mediates the defense response against *Pseudomonas syringae*^56^. Ethylene response factor (ERF) TFs are regulators of pathogenesis-related genes, as well as ethylene-, salicylic acid-, and jasmonic acid-inducible genes^57^. We identified an *ERF9* homologue as a DETF, with induction by *B. xylophilus* inoculation. Results from a previous study indicate that ERF9 is a negative regulator of resistance against necrotrophic fungi and acts as a molecular brake for sustained activation of a defense response^58^. However, it is also plausible that *B. xylophilus* activates expression of *ERF9* homologue to repress the host defense system. Therefore, the DETF families identified here might play roles in fine-tuning of the defense response through regulation of expression of downstream signaling components. In accordance with DETF and GOBP analysis, genes in the *WRKY* and *MYB* TF families, cell-wall biosynthesis, proteolysis, hormone signaling, and defense showed altered expression in Korean red pines inoculated with *B. xylophilus* in a MapMan analysis of the biotic-stress response.

In conclusion, this is the first report that describes the differences in transcriptomes between Korean red pines of felling age inoculated with *B. xylophilus* or *B. thailandae*. Our analysis defined biological processes that are involved in regulation of the defense response against inoculation of *P. densiflora* with *B. xylophilus*. These results might enable the discovery of the genes for the fast identification of *B. xylophilus* infected trees, as well as breeding programs to produce PWD-resistant Korean red pines.

## Supplementary information

Supplementary materials

Dataset 1

Dataset 2

Dataset 3

Dataset 4

## Data Availability

The data generated and analyzed during this study are available from the corresponding author on request.
