# Supplementary material for: Comparative Transcriptome Analysis of Pinus densiflora Following Inoculation with Pathogenic (Bursaphelenchus xylophilus) or Non-pathogenic Nematodes (B. thailandae)
Source: Sci Rep. 2019 Aug 21;9:12180. doi: 10.1038/s41598-019-48660-w (PMC6704138; doi:10.1038/s41598-019-48660-w)
Supplement: Supplementary file 1 — Supplementary materials [file 41598_2019_48660_MOESM1_ESM.pdf]

Comparative Transcriptome Analysis of *Pinus densiflora* Following Inoculation with Pathogenic (*Bursaphelenchus xylophilus*) or Non-pathogenic Nematodes (*B. thailandae*)

Il Hwan Lee[1], Hyerim Han[2], Young Ho Koh[3], In Sik Kim[1], Seok-Woo Lee[1], Donghwan Shim[1]*

[1]Department of Forest Bio-Resources, National Institute of Forest Science, Suwon 16631, Republic of Korea

[2]Division of Forest Insect Pests and Diseases, National Institute of Forest Science, Seoul 02455, Republic of Korea

[3]Ilsong Institute of Life Science, Hallym University, Anyang, Republic of Korea

*Corresponding author (e-mail: shim.donghwan@gmail.com, Tel: +82-031-290-1128, Fax: +82-031-290-1009)

Supplementary Table S1. Oligonucleotide primers used for validation by quantitative reverse-transcription PCR

Gene	Forward primer	Reverse primer
TRINITY_DN124151_c12_g1	TGTGACGAATCCTTCAACGC	AAAGCCGCGGTTTCAAGATC
TRINITY_DN126377_c0_g2	TGCTCCGAACAGTTTGCTTC	TTTTCGAGCCGCCATTAAGG
TRINITY_DN132002_c0_g2	TTCATCACAGCTGCCAATGC	ATGCTCCAGTTTCGTGCATC
TRINITY_DN110089_c0_g1	AATTCACACAGTGCGTCCTG	ACACAATCGACACGGTCTTG
TRINITY_DN127519_c1_g2	AATCCACGACGTGCCAAATG	AGCGCGAAACAAAACCCTAC
TRINITY_DN133087_c0_g4	AAGTCCGTGCATTCTCAACG	TCCGCCATGGAAAATTTGGG
TRINITY_DN132641_c1_g2	AGCGTAGTGAGCGTAAAGGAAG	CCCAGCAAATATACCACACCTG
TRINITY_DN131433_c2_g6	AGAGAAGCAGCAAGAGGAAGAC	TTTGCCGCTGCTTTTGTCTG
TRINITY_DN129047_c0_g2	ACTTGTTGTTGCTGCTGCTG	ACCACGAAAAAGGCAAAGGC
TRINITY_DN129872_c2_g3	AGCTCCTTGTATCGCATTGC	GCTCATTCCGGACAAAACTGC
TRINITY_DN118477_c0_g1	TTTTGCCCCTGAACTGCTAC	TTGCATCCAGAACCGACTTC
TRINITY_DN133874_c0_g1	TGCAAAAAGGACAGCAGCAG	TTTTCGTTTGGGCCAGGTTC
TRINITY_DN130561_c2_g1 (EIF4A-2)	AATGCTTGTCCCACCAACAC	AGTGTCAGGCGCTAGTTTTG

Supplementary Table S2. Statistics for individual trees relating to *Pinus densiflora* RNA sequencing data

Variable	Treatment								
	Water			Non-pathogenic nematode			Pathogenic nematode		
	Water-1	Water-2	Water-3	*B. thailandae* - 1	*B. thailandae* - 2	*B. thailandae* - 3	*B. xylophilus* - 1	*B. xylophilus* - 2	*B. xylophilus* - 3
Yield of raw reads (bp)	6,420,505,764	5,493,374,850	6,068,315,734	6,194,555,836	6,522,800,988	5,518,076,420	6,041,065,732	6,948,199,656	5,629,377,410
Total reads (pairs)	31,784,682	27,194,925	30,041,167	30,666,118	32,291,094	27,317,210	29,906,266	34,397,028	27,868,205
N (%)	0.05	0.05	0.05	0.05	0.05	0.05	0.05	0.05	0.05
GC (%)	48.09	46.30	45.48	45.99	45.30	46.41	46.57	45.53	45.43
Q20 (%)	94.45	94.94	94.56	94.80	94.79	94.78	96.42	96.15	94.85
Q30 (%)	90.48	91.36	90.75	91.15	91.15	91.09	93.83	93.45	91.25
* Q20 (%) – Yield of bases with Q20 or higher.									
Cleaned reads (pairs)	23,040,539	22,981,694	23,424,920	25,097,544	28,088,644	22,702,911	16,860,735	26,189,461	25,309,427
trim_quality	742	573	620	680	696	569	3,103	3,697	635
minimum_length	11,500	9,871	10,699	11,358	11,672	9,942	11,412	13,054	10,187
min_qual_score	84,302	77,543	81,671	85,683	92,266	74,864	72,855	85,338	78,920
min_qual_mean	36,098	26,253	44,231	33,046	30,080	24,698	32,532	49,652	27,760
Dereplication	8,682,103	4,149,593	6,543,747	5,497,760	4,124,882	4,554,149	12,992,800	8,129,244	2,489,932
* Sequences filtered by specified parameters of PRINSEQ-lite.									

Supplementary Table S3. Statistics of predicted types of open reading frame

**Predicted types of open reading frame**	
Complete ORF, *n*	36,184
5′-partial ORF, *n*	13,110
3′-partial ORF, *n*	14,534
Internal ORF, *n*	18,510
Total, *n*	82,338

Supplementary Table S4. Sequencing depth for both unigenes and transcripts

Unigenes			
	Water	*B. thailandae*	*B. xylophilus*
Mean	96.03 ×	97.39 ×	99.43 ×
Median	97.22 ×	99.25 ×	96.79 ×
SE	4.33 ×	4.74 ×	6.24 ×
Transcripts			
	Water	*B. thailandae*	*B. xylophilus*
Mean	37.20 ×	37.73 ×	38.52 ×
Median	37.67 ×	38.45 ×	37.50 ×
SE	1.68 ×	1.84 ×	2.42 ×

Supplementary Table S5. The number and proportion of annotated transcription factors

TF family	No.	Total Number of annotated TFs	Proportion (number of DETFs /total number of indicated TFs)	*P*-value (Fisher's exact test)
WRKY	7	70	0.100	0.049
LBD	6	64	0.094	0.086
bHLH	5	105	0.048	0.572
MYB	5	202	0.025	0.978
ERF	4	112	0.036	0.798
MIKC_MADS	4	42	0.095	0.148
HD-ZIP	1	40	0.025	0.858
CO-like	1	8	0.125	0.342
NAC	1	75	0.013	0.976

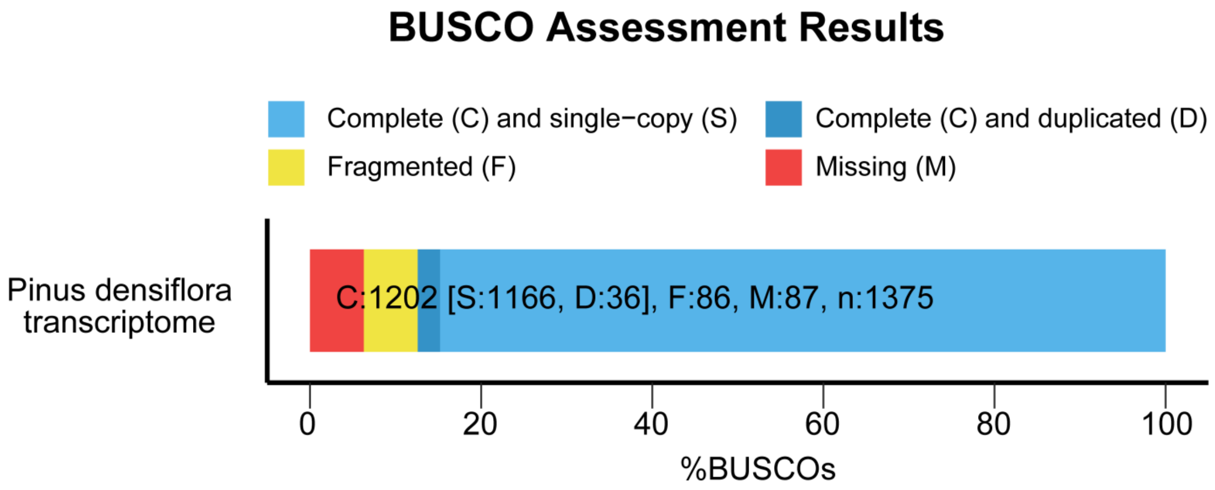

Supplementary Fig. S1. Results of BUSCO analysis of *Pinus densiflora* transcriptome. The number of complete BUSCOs (C), complete and single-copy BUSCOs (S), complete and duplicated BUSCOs (D), fragmented BUSCOs (F), missing BUSCOs (M) and total BUSCO groups searched (n) were shown.

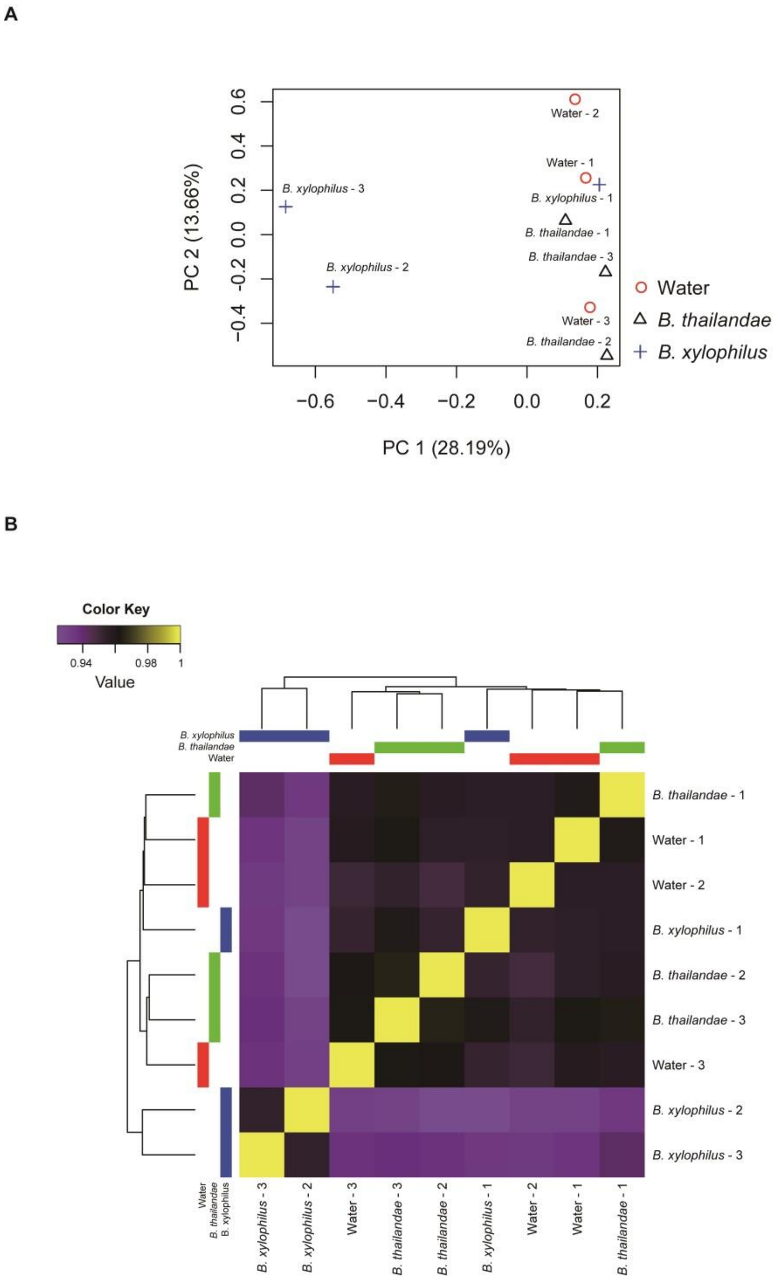

Supplementary Fig. S2. Principal component analysis (PCA) and sample correlation heatmap of RNA-Seq data. (A) PCA plot of all RNA-Seq samples used for the analysis. (B) Clustered heatmap showing the Pearson correlation matrix for pairwise sample comparisons. The color key was adjusted on the basis of the minimal and maximal values for optimal visual differentiation of the differences, and a dendrogram illustrates the relationship–distance between samples. Distance and clustering algorithms used for the dendrogram were complete linkage with Euclidean distances.

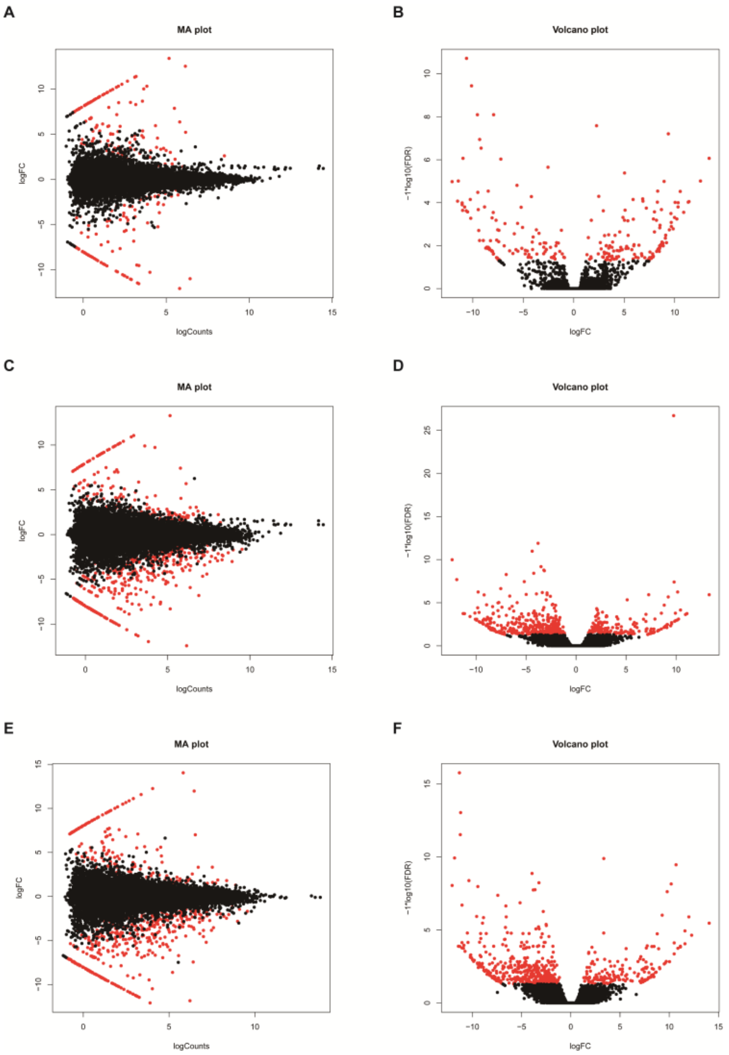

Supplementary Fig. S3. MA and volcano plots for pairwise sample comparisons. MA and volcano plots for pairwise comparisons of expression of transcripts between water and *B. thailandae* inoculation (A and B), between water and *B.* xylophilus inoculation (C and D), and between *B. thailandae* and *B. xylophilus* inoculation (E and F) are shown. For MA plots, Log_2 fold change (logFC) between the two samples is plotted on the *y*-axis and the Log_2 average of the counts normalized by size factor is shown on the x-axis. -Log_{10} FDR is shown on the y-axis and Log_2 fold change (logFC) between the two samples is plotted on the x-axis for volcano plots. The red dots indicate transcripts that were identified as being significant with an FDR $\leq$0.05. These plots were obtained using edgeR in Trinity program.

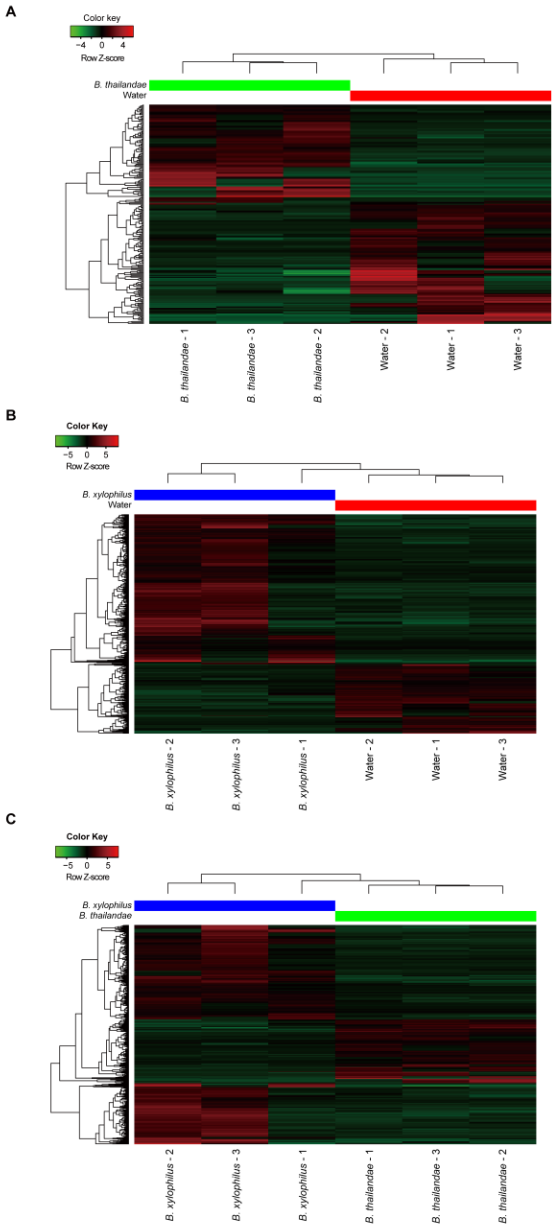

Supplementary Fig. S4. Heatmap of DEGs found in each of the comparisons separately. Heatmaps of DEGs identified in the comparison between water and *B. thailandae* (A), water and *B. xylophilus* (B), *B. xylophilus* and *B. thailandae* (C) are shown. Expression values are log2-transformed median-centred TMM-normalized TPM. Color Key indicates Row Z-scores of expression values. The x-axis dendrogram indicates sample similarity and y-axis dendrogram indicates the hierarchical clustering of unigenes with similar expression profiles. Distance and clustering algorithms used for the dendrogram were complete linkage with Euclidean distances.

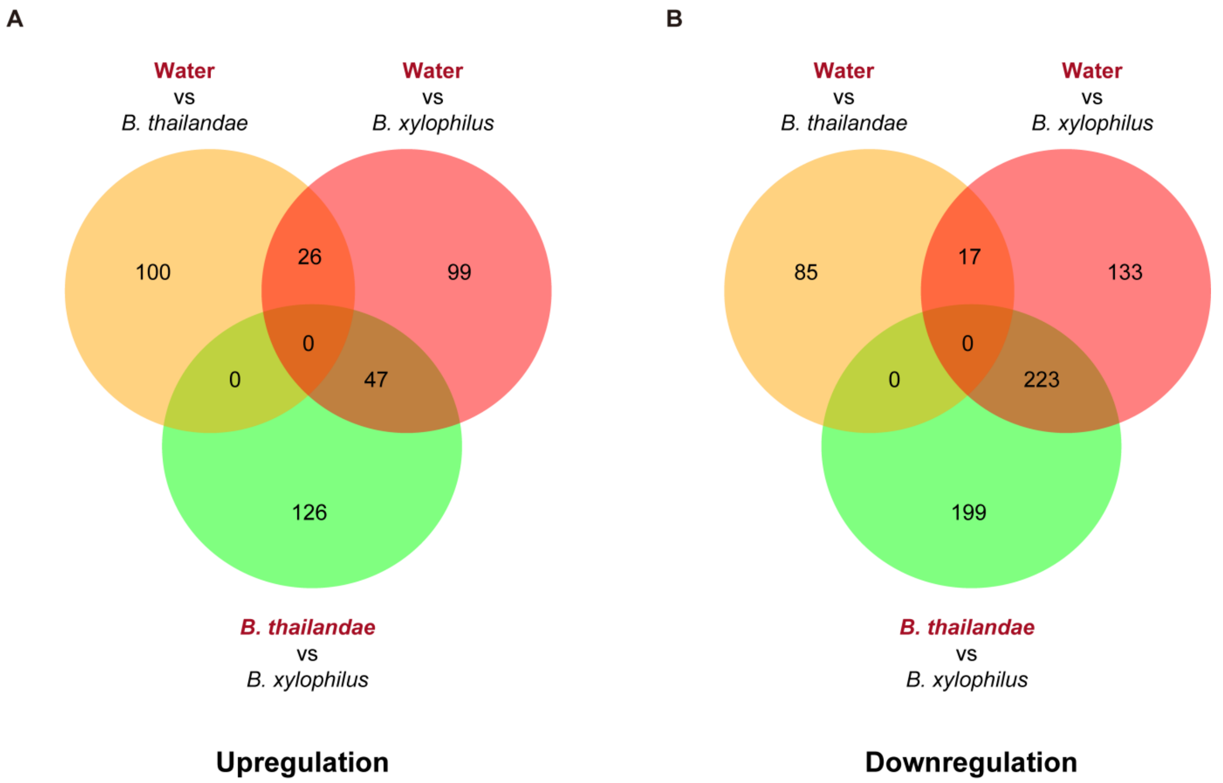

Supplementary Fig. S5. Venn diagrams indicating the numbers of DEGs that showed up and down regulated expression in all pairwise comparisons. The numbers of DEGs showing up (A) and down regulated expression (B) in red colored biological samples at the indicated comparisons are shown.

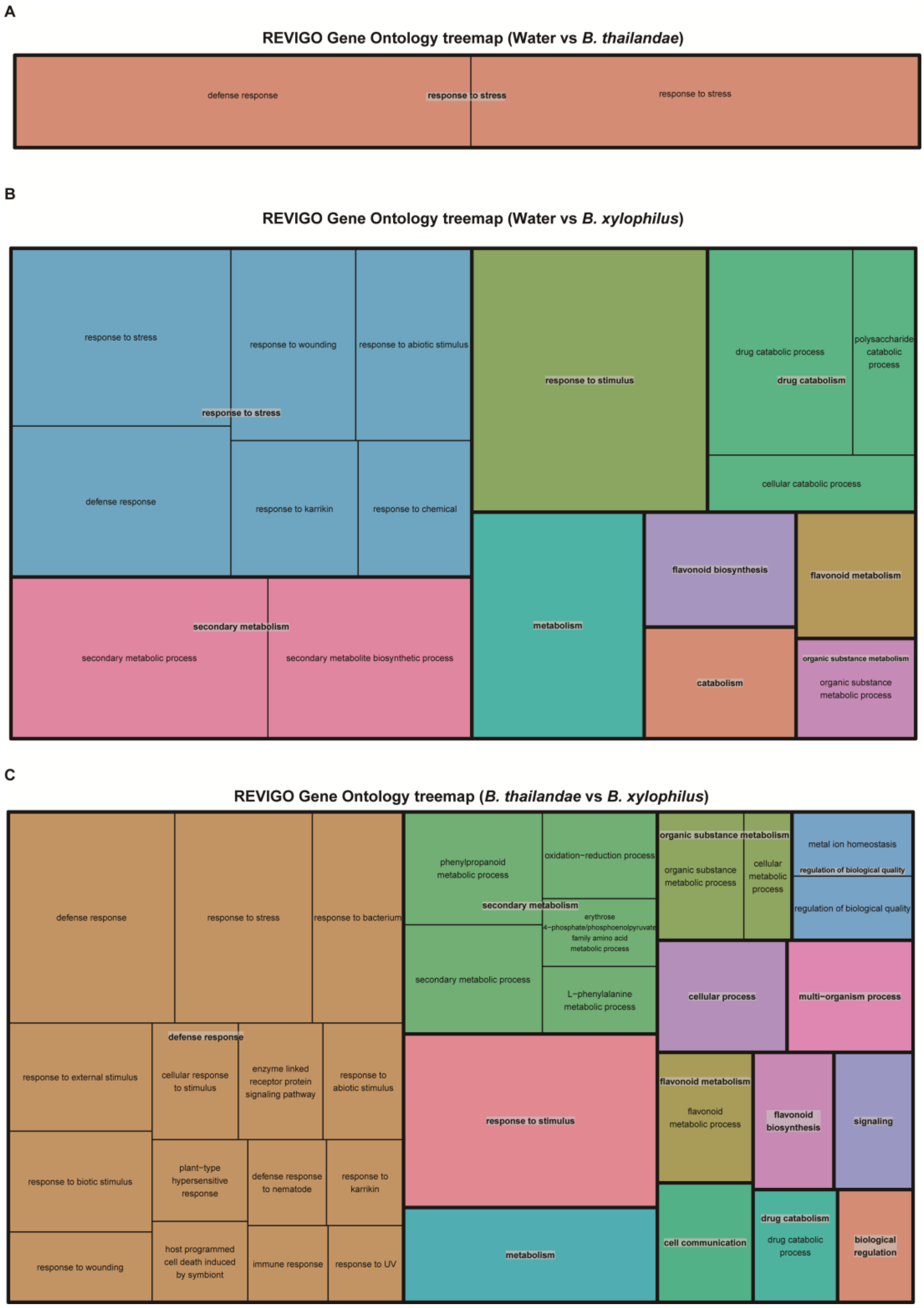

Supplementary Fig. S6. REVIGO treemap of gene ontology (GO) analysis of DEGs. REVIGO treemap showing GOBPs enriched in the DEGs in the comparisons between water and *B. thailandae* (A), water and *B. xylophilus* (B), *B. thailandae* and *B. xylophilus* (C).

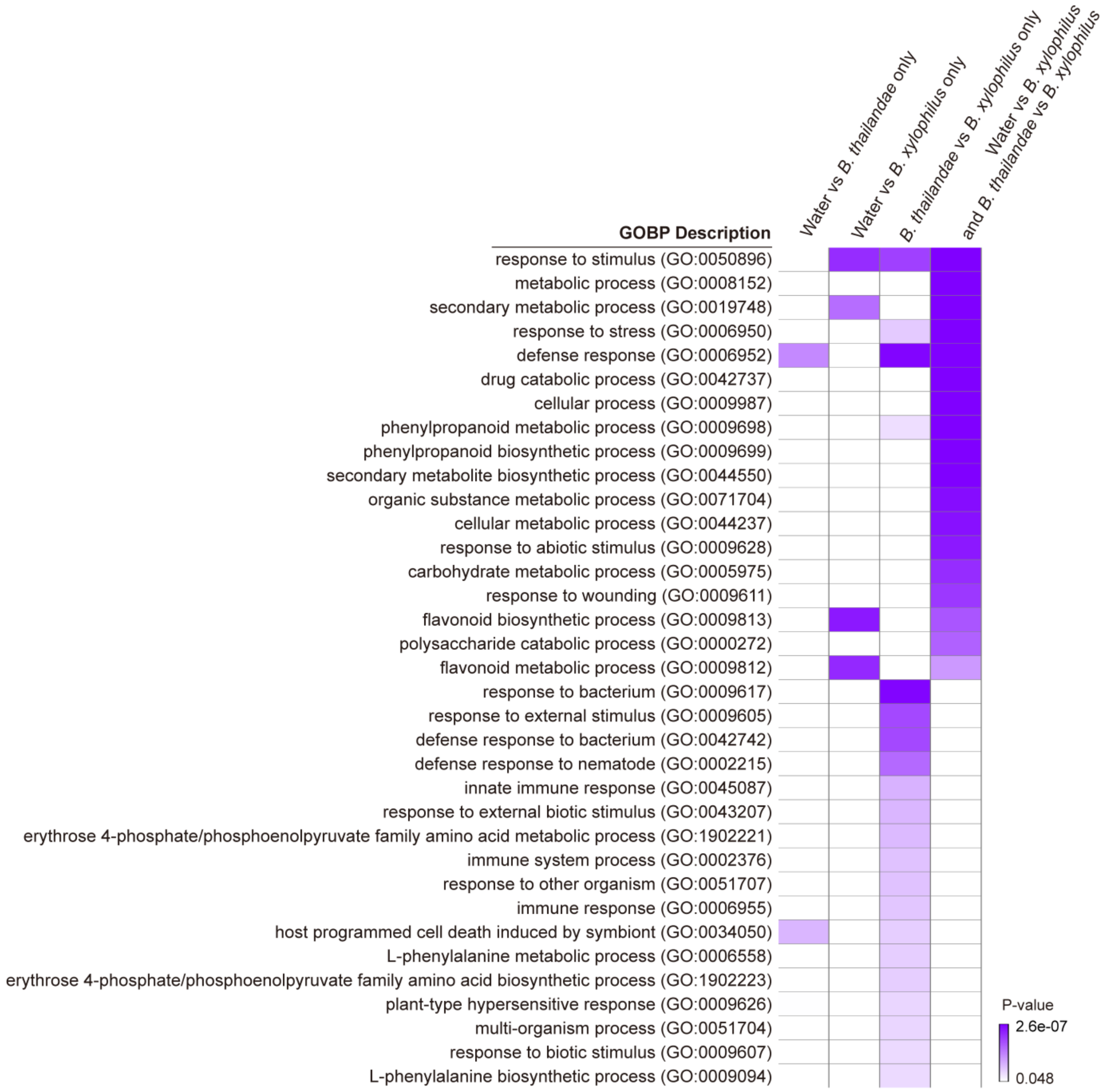

Supplementary Fig. S7. Heatmap of gene ontology (GO) analysis of differentially expressed genes in each category of venn diagram in Fig. 2C. The heatmap shows the GO biological process (GOBP) terms associated with the indicated comparisons (with Fisher's exact test with FDR corrected *P*-value <0.05). Color Key indicates Fisher's exact test with FDR corrected *P*-value .

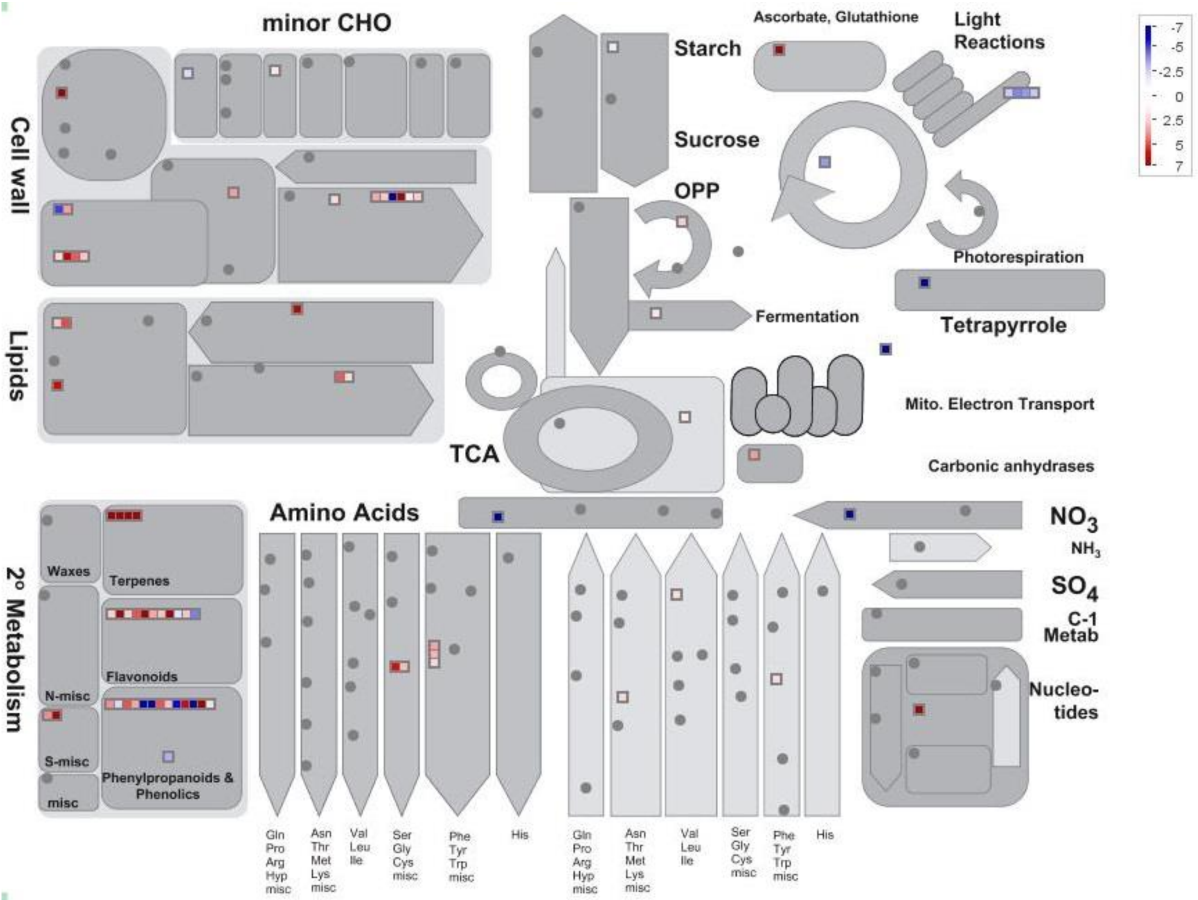

Supplementary Fig. S8. MapMan "Metabolism overview" visualization of *Pinus densiflora* genes involved in the response to *Bursaphelenchus xylophilus* inoculation of Korean red pine trees. Overview of expression levels of DEGs (log$_2$ fold-change of TMM-normalized TPM values) in Korean red pines with *B. xylophilus* inoculation relative to trees with *B. thailandae* inoculation. Square dots represent different paralogous genes encoding proteins that are related to metabolisms in *Arabidopsis thaliana*. Red dots indicate up-regulation and blue dots down-regulation.

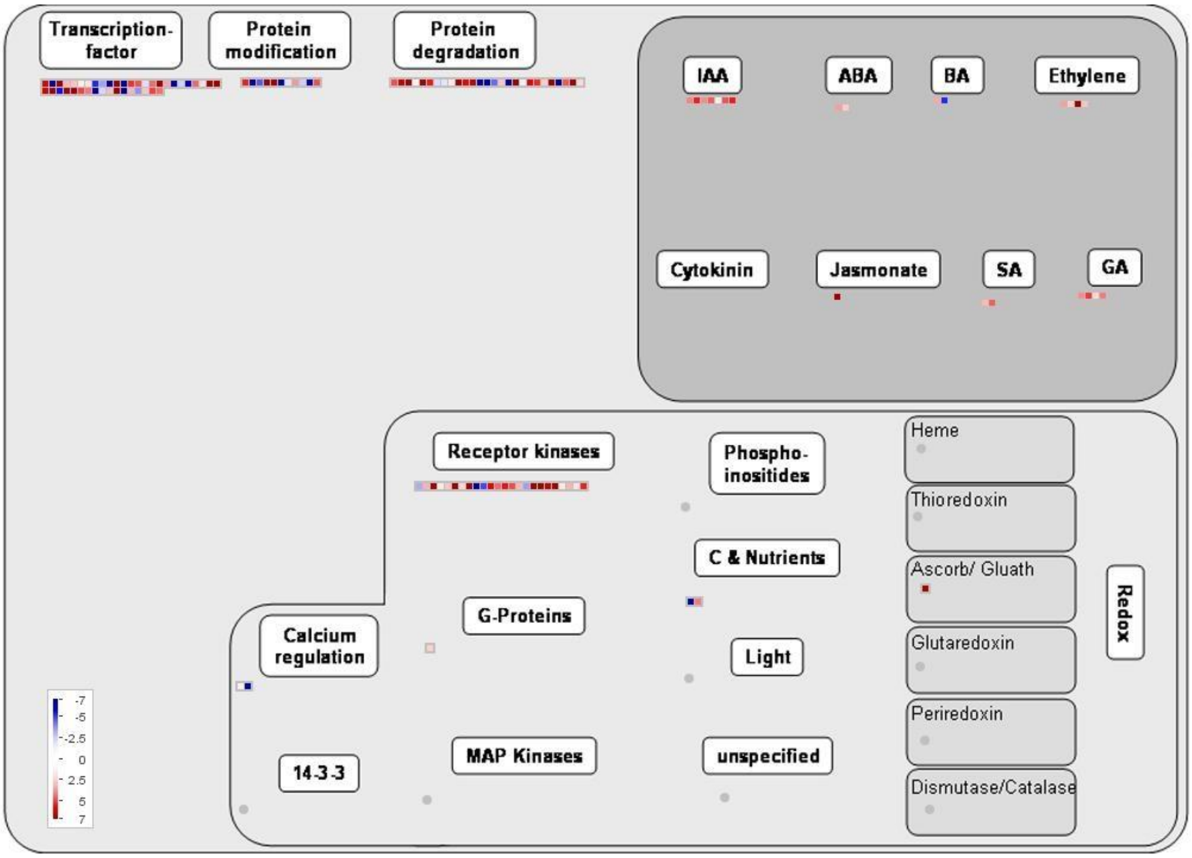

Supplementary Fig. S9. MapMan "Regulation overview" visualization of *Pinus densiflora* genes involved in the response to *Bursaphelenchus xylophilus* inoculation of Korean red pine trees. Overview of expression levels of DEGs ($\log_2$ fold-change of TMM-normalized TPM values) in Korean red pines with *B. xylophilus* inoculation relative to trees with *B. thailandae* inoculation. Square dots represent different paralogous genes encoding proteins that are related to regulation in *Arabidopsis thaliana*. Red dots indicate up-regulation and blue dots down-regulation.

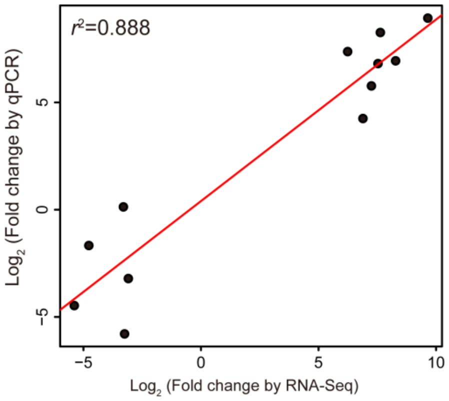

Supplementary Fig. S10. Validation of RNA sequencing (RNA-Seq) results by quantitative PCR (qPCR). Comparison between the $\log_2$-transformed fold-changes in expression levels in Korean red pine trees infected with *Bursaphelenchus thailandae* versus *B. xylophilus*, with data obtained by RNA-Seq and qPCR. The red line indicates the linear relationship between qPCR and RNA-Seq analyses.
