# Supplementary material for: Comparative Transcriptome Analysis of Pinus densiflora Following Inoculation with Pathogenic (Bursaphelenchus xylophilus) or Non-pathogenic Nematodes (B. thailandae)
Source: Sci Rep. 2019 Aug 21;9:12180. doi: 10.1038/s41598-019-48660-w (PMC6704138; doi:10.1038/s41598-019-48660-w)
Supplement: Supplementary file 4 — Dataset 3 [file 41598_2019_48660_MOESM4_ESM.docx]

ID <- c("TRINITY_DN124151_c12_g1",

"TRINITY_DN126377_c0_g2",

"TRINITY_DN132002_c0_g2",

"TRINITY_DN110089_c0_g1",

"TRINITY_DN127519_c1_g2",

"TRINITY_DN133087_c0_g4",

"TRINITY_DN132641_c1_g2",

"TRINITY_DN131433_c2_g6",

"TRINITY_DN129047_c0_g2",

"TRINITY_DN129872_c2_g3",

"TRINITY_DN118477_c0_g1",

"TRINITY_DN133874_c0_g1")

qRT <- c(8.93,

8.26,

6.81,

6.94,

5.77,

7.37,

4.25,

0.13,

-1.67,

-3.21,

-4.47,

-5.79)

NGS <- c(9.65,

7.63,

7.53,

8.28,

7.25,

6.24,

6.89,

-3.30,

-4.77,

-3.09,

-5.39,

-3.25)

validation <- cbind(qRT, NGS)

row.names(validation) <- ID

validation <- as.data.frame(validation)

out=lm(qRT~NGS, data=validation)

summary(out)

plot(qRT~NGS, data=validation)

abline(lm(qRT~NGS, data=validation), col= "red")
